# Adaptation of Imaging Mass Cytometry to Explore the Single Cell Alloimmune Landscape of Liver Transplant Rejection

**DOI:** 10.3389/fimmu.2022.831103

**Published:** 2022-03-31

**Authors:** Nolan Ung, Cameron Goldbeck, Cassandra Man, Julianne Hoeflich, Ren Sun, Arianna Barbetta, Naim Matasci, Jonathan Katz, Jerry S. H. Lee, Shefali Chopra, Shahab Asgharzadeh, Mika Warren, Linda Sher, Rohit Kohli, Omid Akbari, Yuri Genyk, Juliet Emamaullee

**Affiliations:** ^1^ Ellison Institute for Transformative Medicine, University of Southern California, Los Angeles, CA, United States; ^2^ Division of Hepatobiliary and Abdominal Organ Transplant Surgery, Department of Surgery, University of Southern California, Los Angeles, CA, United States; ^3^ Keck School of Medicine, University of Southern California, Los Angeles, CA, United States; ^4^ Department of Chemical Engineering and Material Sciences, University of Southern California, Los Angeles, CA, United States; ^5^ Department of Pathology, University of Southern California, Los Angeles, CA, United States; ^6^ Department of Pediatrics, Children’s Hospital-Los Angeles, Los Angeles, CA, United States; ^7^ Department of Pathology, Children’s Hospital-Los Angeles, Los Angeles, CA, United States; ^8^ Department of Molecular Microbiology and Immunology, University of Southern California, Los Angeles, CA, United States

**Keywords:** single cell analysis, imaging mass cytometry (IMC), CyTOF mass cytometry, allograft rejection, clinical transplantation

## Abstract

Rejection continues to be an important cause of graft loss in solid organ transplantation, but deep exploration of intragraft alloimmunity has been limited by the scarcity of clinical biopsy specimens. Emerging single cell immunoprofiling technologies have shown promise in discerning mechanisms of autoimmunity and cancer immunobiology. Within these applications, Imaging Mass Cytometry (IMC) has been shown to enable highly multiplexed, single cell analysis of immune phenotypes within fixed tissue specimens. In this study, an IMC panel of 10 validated markers was developed to explore the feasibility of IMC in characterizing the immune landscape of chronic rejection (CR) in clinical tissue samples obtained from liver transplant recipients. IMC staining was highly specific and comparable to traditional immunohistochemistry. A single cell segmentation analysis pipeline was developed that enabled detailed visualization and quantification of 109,245 discrete cells, including 30,646 immune cells. Dimensionality reduction identified 11 unique immune subpopulations in CR specimens. Most immune subpopulations were increased and spatially related in CR, including two populations of CD45+/CD3+/CD8+ cytotoxic T-cells and a discrete CD68+ macrophage population, which were not observed in liver with no rejection (NR). Modeling *via* principal component analysis and logistic regression revealed that single cell data can be utilized to construct statistical models with high consistency (Wilcoxon Rank Sum test, p=0.000036). This study highlights the power of IMC to investigate the alloimmune microenvironment at a single cell resolution during clinical rejection episodes. Further validation of IMC has the potential to detect new biomarkers, identify therapeutic targets, and generate patient-specific predictive models of clinical outcomes in solid organ transplantation.

## Introduction

Excellent long-term outcomes can be achieved in solid organ transplantation, in large measure secondary to availability of immunosuppressive drugs that have revolutionized the field (1). However, they are not without side effects, namely renal dysfunction, metabolic syndrome, neurotoxicity, infections, or rarely more serious conditions such as post-transplant lymphoproliferative disease or malignancies, often requiring dose adjustment or even drug discontinuation. In the setting of liver transplantation, (LT), T-cell mediated acute cellular rejection (TCMR) has been reported in up to 30% of LT recipients in the first two years post-transplant, with rates approaching 60% among pediatric LT recipients (1–3). Typically, these rejection episodes can be managed with steroid pulse or lymphocyte depleting strategies, but the long-term effects of these early rejection episodes are not well understood. Approximately 10% of these patients will develop steroid resistant rejection and be at risk for chronic rejection (CR) and late graft loss (4).

The diagnosis of rejection in LT is driven by clinical suspicion based on changes in liver blood tests that prompt an invasive liver biopsy to identify evidence of inflammation and immune infiltrates. TCMR in liver biopsies is assigned a Rejection Activity index (RAI) based on portal inflammation, bile duct inflammation/damage, and venous endothelial inflammation. Histological features which can be seen in chronic rejection include bile duct loss (ductopenia), loss of hepatic arterioles, sinusoidal foam cell accumulation, and cholestasis (5). Despite a long-standing recognition that there is poor correlation between elevation of liver blood tests and active rejection, monitoring these values continues to be the main strategy a clinician can use to suspect a rejection episode after ruling out other causes (6, 7). Indeed, our own recent review of >800 post-LT biopsies, including >150 surveillance biopsies, showed weak correlation between liver biochemistries and rejection (8). Prior studies have attempted to identify new biomarkers of rejection in clinical LT, but these have not been able to deeply examine alloimmunity at the single cell level within the liver, due to technical limitations in working with tiny core biopsy samples [reviewed in (9)]. Recent attempts to identify biomarkers of rejection in post-LT biopsies have used whole genome microarray techniques (10, 11). Some hepatocyte injury related transcription profiles correlated with rejection, but these approaches did not identify specific immune populations to monitor or target therapeutically. These studies highlight the need for single cell analysis of immune-mediated, graft specific processes in LT recipients, using techniques that can overcome limitations of sampling error from biopsy samples and uncover heterogeneous and rare cellular phenotypes that are concealed by population-based measurements (12).

New techniques to characterize immune responses at the single cell level have been developed in recent years. Mass cytometry, or ‘Cytometry by Time-of-Flight’ (CyTOF), utilizes antibodies that are labeled with heavy metal ion tags, with analysis *via* time-of-flight mass spectrometry. The key difference when compared to flow cytometry is that a greater combination of antibody specificities can be analyzed in a single sample (>50), without significant spillover between channels, allowing for deep dissection of complex cellular profiles and relationships (13). The power of CyTOF has recently been expanded to include applications for tissue sections, a process called Imaging Mass Cytometry (IMC) (14). This technique involves labeling a single fixed tissue section with multiple heavy metal ion tagged antibodies (>30) and can overcome nearly all the current limitations of traditional immunohistochemistry, as multiple markers can be studied simultaneously with approximately 1000nm resolution with no requirement for compensation due to autofluorescence. Over the past five years, IMC has been used to deeply analyze the tumor microenvironment, hepatitis B, autoimmune diabetes, and multiple sclerosis in clinical specimens (15–21).

Herein, the potential for IMC to explore alloimmunity in existing clinical FFPE tissue sections from solid organ transplant recipients was explored. An IMC panel was built using validated immune markers, and an analysis pipeline was developed that enabled single cell characterization of histologic relationships between immune subpopulations in clinical LT rejection. Over 100,000 single cells, including >30,000 intrahepatic immune subpopulations, were quantified from clinical specimens to define the immune composition and spatial relationships of alloimmunity in LT and correlated with clinical outcomes.

## Methods

This study was approved by the Health Science Campus Institutional Review Board of the University of Southern California (HS-18-00708) and Children’s Hospital-Los Angeles (CHLA-19-00177).

### Clinical Data and Demographics

Subjects were identified from a prospective institutional transplant database. Patients were included if they had undergone LT alone between 1/2000-12/2018 and then required re-transplant for CR, which was confirmed on explant pathology. All CR tissue used in this study was from the explanted liver. As this was a retrospective study of existing clinical samples, no corresponding serum, frozen tissue, or lymphocytes samples were available for analysis using complementary techniques. A reference population of liver samples with no rejection (NR) was identified by reviewing LT cases for implantation biopsies taken from the donor liver, prior to cross-clamp. Patients were excluded if they had active recurrent viral hepatitis, competing histologic findings (i.e. cholangitis), any evidence of bridging fibrosis, or if there was no clinical tissue block available. A clinical pathologist reviewed each case and identified regions of interest (ROI) that represented relevant diagnostic areas consistent with diagnostic criteria for chronic rejection in liver transplantation (22). Depending on the amount of tissue in the section, the pathologist selected 1-2 1 mm^2^ ROI/patient. Charts were reviewed independently by two members of the research team. Demographics reviewed included age, sex, race, ethnicity, biometrics, primary diagnosis, transplant type, pathology reports, and how rejection episodes were treated (**Table S1**). The population of 18 CR and 5 NR patients resulted in 32 ROI (24 CR/8 NR) used for the analysis.

### Sequential Immunohistochemistry

Formalin fixed, paraffin embedded tissue (FFPE) blocks were pulled from storage at room temperature and cut at 4μm thickness onto Superfrost™ Plus positively charged slides (Thermo Scientific, Kalamazoo, MI), air-dried overnight, and then baked at 70°C for 30 minutes. Slides were loaded onto the Bond Rx Autostainer (Leica Biosystems, Inc, Buffalo Grove, IL) for serial staining with the antibodies (**Table S2**). Antigen retrieval was performed on the Bond Max Rx using either ER1 or ER2 ready to use (RTU) retrieval solution (Leica Biosystems Inc, Buffalo Grove, IL). Antibodies were run using standard Bond Rx protocols with a primary incubation a time of up to 30 minutes. Bound antibodies were stained using Fast Red chromogen (both Leica Biosystems Inc, Buffalo Grove, IL). Between each antibody run, the slides were cover slipped and scanned at 20x using the Leica Aperio™ AT2 (Leica Biosystems Inc) slide scanner. Cover slips were removed using xylene and then alcohol at 100%, 95%, and 75%, sequentially and then de-stained using acid alcohol and acetone sequentially until chromogen disappeared.

### Image Mass Cytometry

The SC2 Core Facility at Children’s Hospital-Los Angeles has developed an immuno-oncology IMC panel for use in neuroblastoma tissue. As this panel includes cancer and immune markers, the subset of validated immune markers available in this panel were used to design our 11-marker IMC panel that would be relevant for liver transplantation (**Table S2**). Collagen and the nuclear intercalator dye were included to provide morphometric data and facilitate single cell segmentation. FFPE sections (4μm thickness) were stained with isotope-conjugated antibodies (**Table S2**) for imaging according to the protocol offered by Fluidigm (PN 400322 A3) with minor modifications. In brief, sections were baked at 60°C for 2 hours, dewaxed in xylene for 20 minutes, hydrated with alcohol, and washed in 18.2MΩ water for 5 minutes. They were then incubated in preheated antigen retrieval solution (Dako, S2367) for 30 minutes at 100°C. Following incubation, the antigen retrieval solution was allowed to sit at room temperature for 10 minutes, and then slides were washed with 18.2MΩ water followed by a wash in Maxpar phosphate buffered saline (PBS, Fluidigm, 201058). Tissue sections were then blocked for 45 minutes with 3% bovine serum albumin and incubated overnight with the IMC antibody panel. Next, slides were washed with Maxpar PBS, 0.1% Triton X-100 (Thermo Scientific, 85111). Samples were then incubated with 1:800 intercalator-Ir (Fluidigm, 201192A) in Maxpar PBS for 45 minutes followed by a 4-minute wash with Maxpar PBS. Finally, sections were stained with 0.0005% RuO4 (Electron Microscopy Sciences) solution for 3 minutes, washed with 18.2MΩ water for 10 seconds, and allowed to dry. ROI as indicated by the clinical pathologist were selected. Stained tissue slides were ablated using the Hyperion Imaging System (Fluidigm) at a laser frequency of 200Hz with a power range of 3.5-4.5. The ablation procedure produced.txt and.mcd files which were used in analysis (23).

### Data Transformation, Processing, and Segmentation

All IMC data are based on raw measurements and were not transformed. Utilizing the ‘ImcSegmentationPipeline’ repository on Github created by the Bodenmiller Lab as a guide, high quality image segmentation was performed on ROI from each tissue section (24). Supplying the open-source pipeline with zipped folders of raw IMC data in the.mcd and.txt formats, the data was converted into the ome.tiff format in order to generate two analysis stacks of.tiff format images: the ‘Full’ stack, of all channels of interest and the ‘Ilastik’ stack, which only includes channels necessary for cell structure segmentation. In CellProfiler Ver. 3.1.8, the pipeline ‘1_ilastik_preprocessing’ was used to prepare the images within the stack by removing outlier pixels to reduce noise, scaling the image 2x, and cropping random sections to be used in model training (16). Image crops were then loaded into Ilastik Ver. 1.3.2 to manually train the pixel classifier to distinguish three classes of pixels: nuclear, cytoplasmic/membrane, and background. The ‘Batch processing’ function was used to convert the Ilastik stacks into probability maps (25). In CellProfiler, the probability map stack is used to segment nuclei and single cell masks using pipeline ‘2_segment_ilastik’ while rescaling the image to the original 1x resolution. These masks and the ‘Full’ stack were loaded into CellProfiler pipeline ‘3_measure_mask’ to generate intensity and single-cell data. Raw and single cell segmentation data were visualized in HistoCAT v.1.76 or R (23).

### IMC Data Visualization

IMC.mcd files were loaded into HistoCAT v.1.76 for visualization. Raw data were examined directly and pseudocolored to identify individual markers. Immune meta-clusters were verified on individual ROI by selecting specific cluster IDs from Phenograph analysis and pseudocolored to identify specific cells on each tissue section using Histocat v1.76, and R using the ggplot2 package v3.3.2 (26, 27).

### Barnes-Hut tSNE and Phenograph Clustering

Unsupervised clustering and aggregation of single cell data from the entire study population was completed using t-distributed stochastic neighbor embedding (tSNE) transformation, a non-linear dimensionality reduction useful for high-dimensional data, and the Phenograph function in Histocat v1.76. Outputs of Phenograph, a graph-based algorithm, were exported and analyzed further in R. Default tSNE parameters (initial dimensions: 110, perplexity, 30, theta, 0.5) and Phenograph (nearest neighbors of 75) within Histocat were used (23). Specific meta-clusters were visualized by overlaying tSNE plots with Phenograph outputs in Histocat. Meta-clusters were plotted against standardized individual immune markers using a heatmap to establish an identity for each meta-cluster based on marker expression and established association with specific immune subsets.

### Neighborhood Analysis

Spatial, single cell pairwise neighbor interactions (relative proximity and avoidance) between Phenograph meta-clusters were examined using the validated neighborhood analysis function in Histocat v1.76. In this initial Histocat-based analysis, a neighboring cell was defined as a cell located within 4 pixels (99 permutations, p<0.05) of a specific Phenograph meta-cluster of interest. This analysis measures pairwise interactions between and within cellular phenotypes *via* comparison to random cellular distribution using two individual one-tailed permutation tests, which then allows for calculation of a p-value indicating either significant interaction or spatial avoidance. For more in-depth analysis of immune cluster interactions, neighbouRhood v0.3.0 was used in R. Single cell and object relationship data was used from Cell Profiler and combined with the marker information from Histocat output, and the following analysis settings were applied: 5000 permutations, 4-pixel radius, and p-value of <0.01. The neighborhood analysis was executed in and visualized in R using the neighbouRhood v0.3.0 and ggplots v3.1.0 packages to identify interactions between immune subpopulations.

### Statistical Analysis

Average marker signal intensity was calculated as the median signal across all cells for a given marker per ROI; differences between study populations were tested using the Wilcoxon Rank-Sum test. Immune meta-cluster proportions were calculated, excluding non-immune cells, as the total number of cells of a given subpopulation divided by the total number of immune cells per ROI; differences between study populations were tested using the Wilcoxon Rank-Sum test. Additionally, the number of cells in each meta-cluster were counted per ROI and standardized within meta-cluster to be visualized across all ROIs. Principal Component Analysis (PCA) was performed in a downstream analysis on the median marker signal and immune meta-cluster cell count per ROI; whereas tSNE is a stochastic technique, PCA is deterministic and therefore easily replicated. Once decomposed, leading Principal Components (PCs) were extracted to determine variance explained, compare CR and NR along PC paths, as well as identify marker composition. Finally, the leading PC was put into a Logistic Regression model predicting Chronic Rejection. A correlation network across IMC panel markers was also created to visually assess interactions (**Figure S1**). A p<0.05 was considered significant, and all statistics were performed in R. All non-histologic figures were created in R.

## Results

### Patient Characteristics

Patients with a history of LT that underwent re-transplantation at our center for CR over a period of 20 years were selected. Given the history of re-transplantation, the entire liver allograft was explanted, and as a result, multiple tissue blocks and multiple ROI per slide were available from the same liver explant for technical optimization of the IMC technique and downstream analysis. Cases were included following review if the primary explant diagnosis was CR with ductopenia, with no evidence of confounding diagnoses (i.e. cholangitis) or any bridging fibrosis. A total of 18 patients met inclusion criteria (**Table 1**). The population was predominantly Caucasian (94.4%) and Hispanic (55.5%), and 61.1% were male. Most patients received steroid induction for the first transplant, and all patients had at least one episode of biopsy-proven TCMR and/or CR prior to re-transplant (range 1-7; **Table S1**). All patients had developed CR despite being treated with pulse steroids at least once prior to re-transplant, and only one patient had documented donor-specific alloantibody, with no evidence of antibody mediated rejection. The median time interval between the first LT and re-transplant for CR was 2 years (IQR: 0.6-11.4). To generate a NR control group, LT cases were reviewed to identify wedge liver biopsies taken prior to cross-clamp, and pathology reports were reviewed to confirm normal histologic appearance. Five NR liver specimens were identified, and demographics are outlined in **Table 1**.

**Table 1:** Patient characteristics.

Patients with chronic rejection	Total patients: 18
Age at re-transplant, years, median [IQR]	34.5 [23,50.8]
Interval between 1^st^ and 2^nd^ liver transplant, years (median [IQR])	2 [0.6,11.4]
Sex, no. male (%)	11 (61.1)
Race	
Caucasian, N (%)	17 (94.4)
Asian, N (%)	1 (5.5)
Black, N (%)	0
Ethnicity	
Hispanic, N (%)	10 (55.5)
Primary Etiology of Liver Disease requiring LT	
Viral hepatitis, N (%)	6 (33.3)
Alcohol use disorder, N (%)	2 (11.1)
Acute liver failure, N (%)	4 (22.2)
Biliary atresia, N (%)	2 (11.1)
Autoimmune hepatitis, N (%)	1 (5.5)
Metabolic Disorder, N (%)	2 (11.1)
Wilson’s Disease, N %)	1 (5.5)
Induction immunotherapy (First transplant)	
Steroids, N (%)	15 (83.3)
Steroids + Anti-CD25, N (%)	2 (11.1)
Anti-CD25, N (%)	1 (5.5)
Rejection episodes prior to re-transplantation, number (median [IQR])	2 [1,3] Range 1-7
Patients with no rejection	**Total patients: 5**
Age, years, median [IQR]	53 [51,55]
Sex, no. male (%)	2 (40.0)
Race	
Caucasian, N (%)	4 (80.0)
Asian, N (%)	1 (20.0)
Black, N (%)	0 (0)
Ethnicity	
Hispanic, N (%)	2 (40.0)

### IMC Panel Creation and Validation in Clinical Liver Specimens

At the outset of this study, our CyTOF core facility had independently optimized and validated 22 unique isotope-tagged antibodies for use in IMC on human tissue, composed primarily of oncology and immune markers. From this panel, 8 immune markers potentially relevant to rejection were selected: CD20 (B-cells), CD68 (macrophages), CD66a (neutrophils), CD45 (pan-leukocyte marker), CD45RA (naïve T-cells), CD3 (T-cell receptor), CD8 [Cytotoxic T-cells (CTL)] and HLA-DR. Collagen-1 was selected as a non-immune marker to enhance visualization of tissue architecture, as collagen, which is typically detected using Masson’s Trichrome staining in clinical liver biopsies, is expressed in the portal triads of healthy liver and increases during fibrosis (28). A nuclear intercalator dye (Iridium) was included to facilitate identification of individual cells and segmentation in the analysis pipeline. Thus, there were 9 IMC antibodies and one nuclear stain for simultaneous visualization on each tissue section.

**Figure 1:**
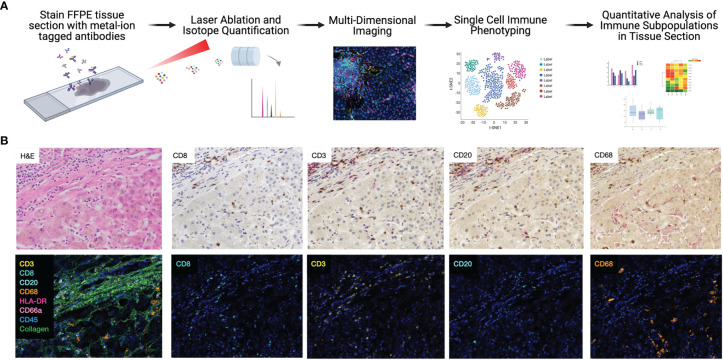
Highly dimensional, single-cell immune phenotyping of human liver tissue with IMC. **(A)** Schematic of IMC data acquisition and analysis pipeline developed for this study. **(B)** Individual immune markers in FFPE clinical liver samples were examined using sequential immunohistochemistry (top panels; representative patient and subset of markers shown) and IMC (lower panels, adjacent tissue section from the same patient is shown to maintain morphological features).

First, each IMC marker was validated independently by comparing to traditional immunohistochemistry staining, though previous studies have clearly established the comparability and reproducibility of IMC (15, 17, 23). One of the major strengths of the IMC technique is the opportunity to stain many markers simultaneously on a single section. As a corollary, sequential immunohistochemistry has been developed to allow for repeated single antibody staining on a single tissue section, with commercially available automated devices for use in clinical labs. To compare the integrity and distribution of IMC markers with traditional immunohistochemistry, sequential sections from the same block were stained with each technique, for each marker using a similar strategy to a recent diabetes study (17). Representative images from both IMC and immunohistochemistry from a single tissue block are shown in **Figure 1B**. Traditional immunohistochemistry identified each marker, but sequential staining resulted in a progressive increase in background staining. Each marker in our IMC panel stained appropriately, and very specifically, in a similar pattern to sequential immunohistochemistry without any background.

**Figure 2:**
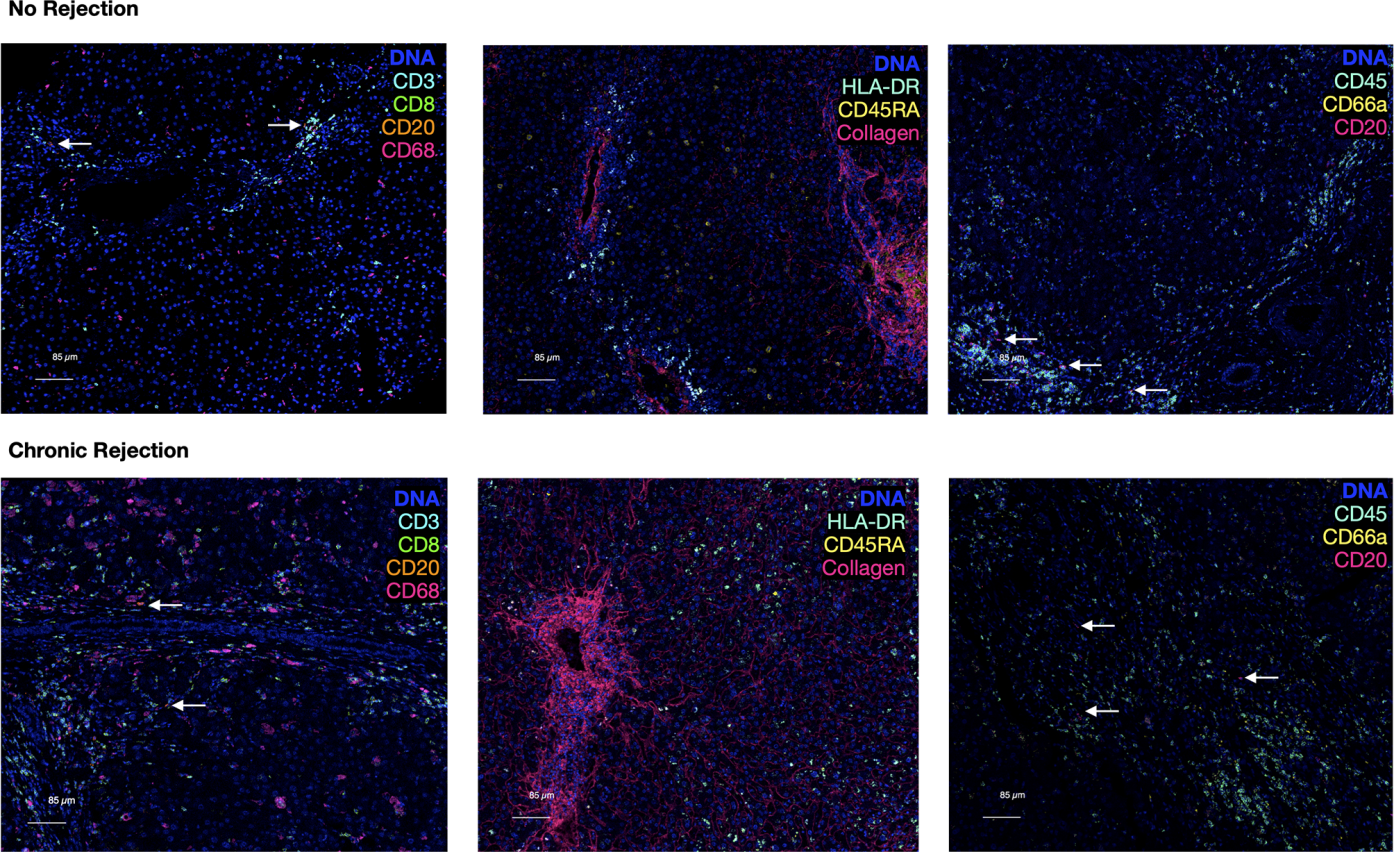
Representative pseudocolored images of IMC markers in liver with no rejection versus chronic rejection post-transplant. Antigens targeted by the isotope-conjugated antibodies of the 10 marker IMC panel that was used to stain liver tissue with no rejection and liver transplant recipients with chronic rejection. These are representative IMC images from the analyzed cohorts generated by IMC. A DNA intercalator dye (iridium) was used to identify nuclei. White arrows indicate rare CD20+ cells.

Comparison of CD20 staining, for example, reveals residual CD3 staining with sequential immunohistochemistry, whereas IMC resulted in highly specific CD20 staining with no background. The median signal per marker, per ROI, was calculated and compared between NR and CR (**Figure S2**). This analysis was not based on single cell resolution and highlights that IMC staining intensity for each marker is quantitative rather than qualitative and highly discrete, but some heavy metal ion tagged antibodies result in more signal intensity than others. Representative pseudocolored images of IMC staining on three unique patients from the NR and CR cohorts are shown in **Figure 2**. As an example, CD20+ B-cells were both rare and stained with less brightness than CD8 for both NR and CR, resulting in less median signal (white arrows indicate CD20+ cells; **Figure 2**). Importantly, the tissue blocks included in this study had been stored in the clinical pathology lab, at room temperature, for 0.5-20 years, with no apparent degradation in quality of staining using IMC.

### Single Cell Analysis Enables Identification of 11 Unique Immune Subpopulations Involved in CR

**Figure 3:**
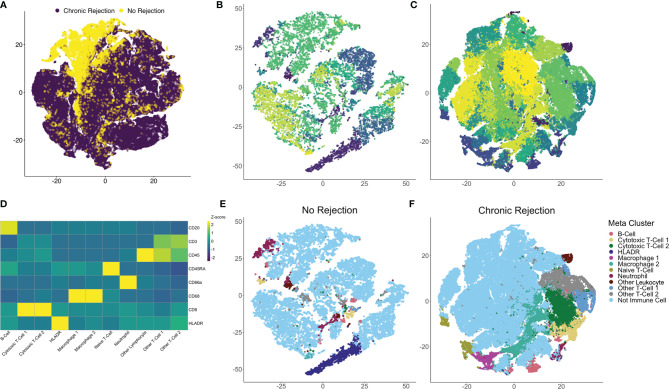
Identification of individual immune subpopulations present in high-dimension histopathological analysis of clinical liver transplant rejection. **(A)** T-distributed stochastic neighbor embedding (tSNE) plots of 109,245 individual cells identified using IMC of liver with no rejection (purple, 16,454 cells) and chronic rejection (yellow, 92,791 cells) were created to compare the two patient cohorts, revealing global differences in cellular meta-clusters. Next, Phenograph plots were created for cells identified in liver tissue to identify immune meta-clusters. Among 29 total cellular meta-clusters identified through the combined dataset of liver with no rejection **(B)** versus chronic rejection **(C)**, 11 had at least one immune marker present. Immune meta-clusters were then applied back to tSNE plots for no rejection **(E)** and chronic rejection **(F)** to visualize density and distribution of immune subpopulations of interest. To further characterize these immune subpopulations, a heatmap of column-standardized median marker intensity for each immune meta-cluster was created to associate individual markers with cellular phenotypes (panel D, color scale represents z-scores for each marker).

To quantify each marker, we developed an IMC segmentation and analysis pipeline based on the ‘IMC Segmentation Pipeline’ repository created by the Bodenmiller group (23, 24, 29). Ilastik and CellProfiler were used to prepare the images for segmentation based on an overlay of all markers to identify cytoplasm/membrane, nuclei (iridium), and non-cellular space. This step is independent of signal intensity for markers, as it captures overall cellular morphometry. One of the strengths of single cell segmentation of liver tissue, when compared to cancer tissue, is the relatively uniformity of cell morphology, which resulted in high-quality and reproducible segmentation. These data were subsequently loaded into HistoCAT for quantification and downstream analysis of single cell populations. Once IMC outputs have been segmented into a single-cell dataset, differentiation of immune subpopulations can be completed using a similar strategy to flow cytometry. Following segmentation, 109,245 individual cells were identified from NR (16,454 cells) and CR (92,791 cells). Dimensionality reduction using the tSNE algorithm allowed for visualization of the highly multiplexed, single cell dataset in two dimensions. tSNE was selected as it has been most widely used for clustering of IMC data, rather than other techniques such as UMAP (15, 23, 30). Comparison of CR and NR *via* tSNE highlighted global differences in the distribution and grouping of single cell data (**Figure 3A**). Next, Phenograph analysis, which is a nearest neighbor graph based method, was applied to the entire dataset using IMC markers to identify distinct subpopulations based on marker distribution, signal intensity, and cellular morphology (31). This identified 29 distinct meta-clusters, among which 11 (30,646 cells) were associated with at least one of the 8 immune markers in the IMC panel (**Figure 3B**: NR, **Figure 3C**: CR). The remaining 18 non-immune meta-clusters were identified based on nuclear staining, collagen, and morphology and combined into one category ‘not immune cell’. A heatmap plot visualizing relative signal intensity per cell by individual marker enabled phenotypic classification of the 11 immune-related meta-clusters (**Figure 3D**). Of note, one of the caveats to performing IMC is that the baseline signal intensity for a specific marker is still dependent on the quality of the antibody and its overall performance in immunohistochemistry, whereas antibody signal intensity in flow cytometry or CyTOF is generally proportional to antigen density and can be compared across antibodies. That being said, within an antibody marker, IMC is still semi-quantitative. Thus, if a panel of slides is stained using IMC for a discrete marker, the intensity of that marker across cells in an individual section and across different ROI obtained from that same type of tissue, stained using the same method, can be measured and compared. The same cannot necessarily be inferred when comparing two or more different markers. Thus we do not call our cells ‘CD3^hi^’ or ‘CD3^low^’ but rather classify them based on the Phenograph clustering algorithm as simply CD3+ or CD3- and label unique Phenograph clusters with similar markers but different clustering properties as ‘population-1’, ‘population-2’ etc.

Using this approach, we confirmed that Phenograph meta-cluster marker outputs were associated with appropriate distributions of immune markers (i.e. no CD3 expression on CD68+ macrophages). Interestingly, not all T- or B-cell populations were strongly CD45+, which has been observed by other groups using the IMC technique (15, 19–21). Immune meta-clusters based on solitary markers included B-cells (CD20+), HLADR+ cells, Naïve T-cells (CD45RA+), Other Leukocytes (CD45+), and Neutrophils (CD66a+). Phenograph clustering also identified unique immune subpopulations with the same markers but different clustering based on signal intensity and cellular morphology, i.e. two distinct CD45+CD3+CD8+ CTL meta-clusters (CTL-1 and CTL-2), two distinct CD68+ macrophage populations (Macrophage-1 and Macrophage-2), and two unspecified CD45+CD3+ T-cell populations (Other T-cell-1 and Other T-Cell-2). The 11 immune subpopulations identified with this approach were visualized on tSNE plots for NR (**Figure 3E**) and CR (**Figure 3F**), confirming the unique identity and localization of each immune meta-cluster.

**Figure 4:**
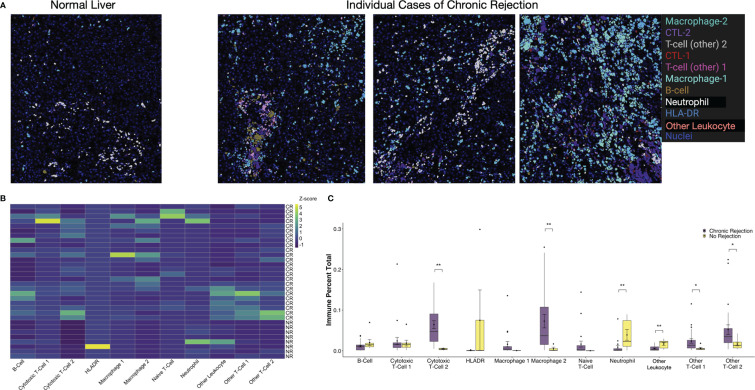
Visualization and quantification of immune subpopulations involved in chronic liver transplant rejection. **(A)** Histopathological examination of immune subpopulations identified *via* Phenograph analysis was completed by creating single-cell masks labeled by specific immune cell meta-cluster. Representative images depict liver with no rejection and three unique patients with chronic rejection. **(B)** The relative frequency of specific immune subpopulations in individual regions of interest for no rejection (NR) and chronic rejection (CR) across the study population standardized within subpopulation using z-scores, where the zero-point is the average cell frequency within that subpopulation. Color scale shows z-score per column. **(C)** Quantification of each immune subpopulation revealed significant increases in the following subpopulations in chronic rejection when compared to liver with no rejection: Cytotoxic T-cell 2, Granulocytes, Macrophage 2, Other T-cell 1 and Other T-cell 2 (*p < 0.05, **p < 0.01 for comparisons between NR and CR).

Another unique aspect of IMC is the ability to create masks on a per cell basis based on Phenograph meta-clusters. Typically, for multiplexed immunofluorescence, fluorochromes are selected such that co-localization of individual markers results in a combined color, i.e. red and green fluorescence overlay to produce a yellow signal. With the dimensionality of IMC data, specific Phenograph clusters can be labeled discretely and localized on tissue sections in Histocat. The 11 immune subpopulations identified were visualized on individual ROI from both NR and CR (**Figure 4**). Clinically, a diagnostic criterion for CR is the presence of inflammation, which is identified on tissue sections as areas with immune infiltrates. By visualizing specific immune meta-clusters on tissue sections, the IMC technique reveals the identity of these cells, which involve a heterogenous conglomeration of macrophages, T-cells, and rare B-cells (**Figure 4**).

### Quantitative and Spatial Analysis Highlights Significant Interactions Between Immune Subpopulations Associated With CR

Single cell segmentation of IMC data also allows for both quantitative measurements of differences between patient cohorts and statistical analysis of spatial relationships, including interactions and avoidances among various Phenograph meta-clusters on tissue sections (16, 23). Among the 11 immune subpopulations (30,646 cells) identified, 91.1% (27,908 cells) were present in CR and only 16.6% (2,738 cells) were present in NR. To account for the different number of samples and total immune cells between CR and NR, comparison of each immune subpopulation was examined by standardization with a z-score, where the zero-point is the average cell frequency within that subpopulation, to compare the frequency of each immune meta-cluster across individual patients (**Figure 4B**). We also examined the relative proportion of total immune cells in each patient cohort (**Figure 4C**). When compared to NR, liver sections with CR had increased proportions of Macrophage-2 (p<0.01), CTL-2 (p<0.01), other T-cell-1 (p<0.05), and other T-cell-2 (p<0.05) populations (**Figure 4C**). Indeed, Macrophage-2 was essentially absent in NR. On the other hand, CD66+ neutrophils (p<0.01) and Other Leukocytes (CD45+, p<0.01) represented a greater proportion of immune cells in NR when compared to CR.

**Figure 5:**
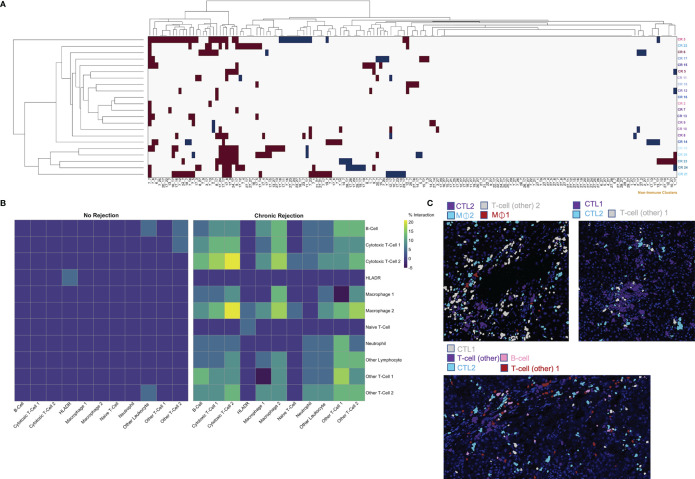
Characterization of spatial relationships between immune subpopulations mediating chronic liver transplant rejection. Phenograph meta-cluster data, including non-immune populations, were examined using neighborhood analysis, which identifies significant, pairwise interactions or avoidances between individual cells within a 4-pixel (4 μm) radius. **(A)** Clusterogram depicting individual ROI from patients with chronic rejection across each row, highlighting significant pairwise interactions (red) and avoidances (blue) based on cluster ID across all 29 Phenograph clusters, including 18 unique ‘non-immune cell’ clusters, enables visualization of significant interactions and avoidances across the entire dataset (Histocat, 99 permutations, p<0.05). **(B)** This visual representation was further narrowed to focus exclusively on immune subpopulations using neighbouRhood in R (5000 permutations/p<0.01). Immune cell interactions were rare in liver with no rejection, while several strong interactions were noted in chronic rejection. Color scale shows percentage of significant interaction events. **(C)** Representative images were pseudocolored to highlight immune populations with significant interactions, including Cytotoxic T-cell-2 with Macrophage-1 and Macrophage-2, as well as interactions between Cytotoxic T-cell-1 and Cytotoxic T-cell-2.

The ‘neighborhood analysis’ feature of Histocat examines spatial pairwise interactions and avoidances between Phenograph meta-clusters (15, 23). We first used the Bodenmiller/Histocat approach to perform a qualitative examination of interactions across all 29 Phenograph meta-clusters, including 18 ‘non-immune cell’ clusters (23). This approach highlights significant interactions in red and avoidances in blue (p<0.05). Individual patients with CR demonstrated multiple significant interactions (burgundy boxes in Histocat Clusterogram plot, 99 permutations, p<0.05, **Figure 5A**), generally among immune meta-clusters (cluster ID in **Table S3**), with rare avoidances, particularly among non-immune clusters (blue boxes, right side of panel A). A more detailed and quantitative neighborhood analysis was conducted specifically focusing on immune subpopulations using the NeighbouRhood package in R (5000 permutations, p<0.01), and the percent of significant immune cell interactions was quantified and visualized on a heatmap (**Figure 5B**). A significant interaction was given a positive value, and an avoidance was given a negative value, such that a single positive and negative interaction would cancel each other, based on p<0.01. Significant interactions or avoidances were rare in NR (5/121 possibilities; 4.1%), which is consistent with imaging findings of sparse and isolated immune subpopulations (**Figures 4A** and **5B**). Conversely, 78 strong interactions were observed in patients with CR, and the strongest were noted among CTL-1 and CTL-2, Macrophage-1 and CTL-2, Macrophage-2 and CTL-2, and Other T-cell-1, Other T-cell-2, and B-cells (**Figure 5B**). These immune cell interactions correlate well with the observed upregulated populations observed in CR in **Figure 4C**. Pairwise significant interactions were visualized on tissue sections by selecting Phenograph meta-clusters, and representative tissue sections are shown in panel 5C, confirming that immune populations that have significant interaction are often touching one another. Collectively, these data suggest a coordinated, complex immune response during chronic allograft rejection.

### Modeling IMC Data Enables Correlation With Clinical Transplant Outcomes

**Figure 6:**
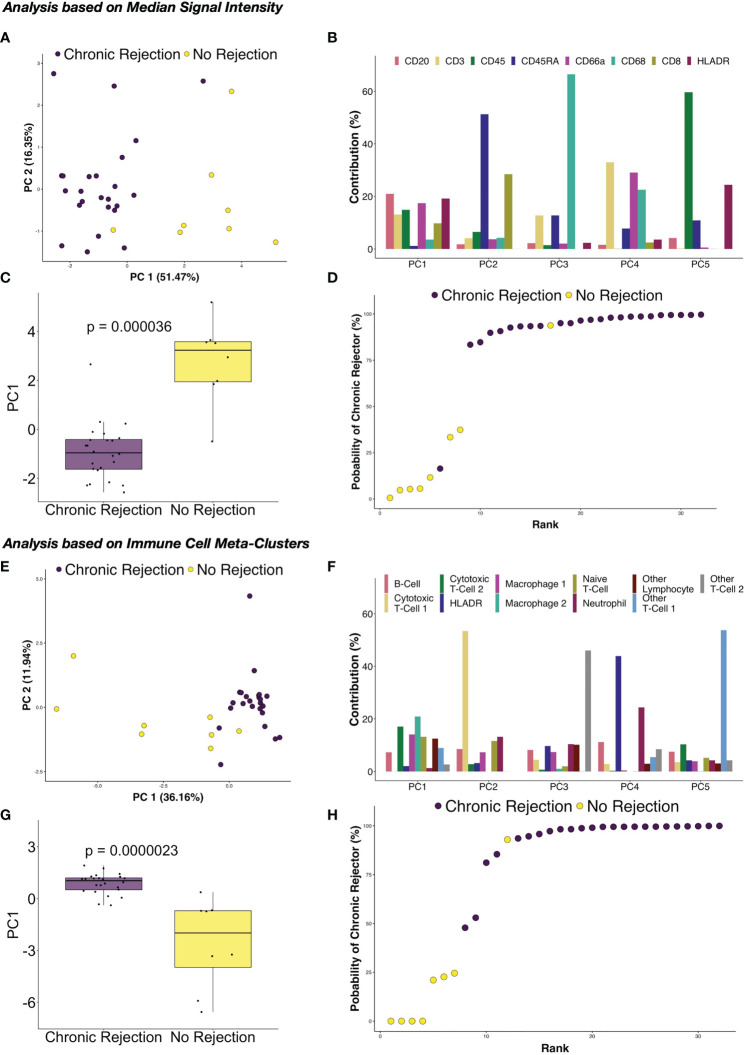
Principal component analysis of immune marker distribution *via* IMC demonstrates strong correlations with chronic rejection in clinical liver biopsies. Following dimensionality reduction, tSNE plots obtained from individual regions of interest suggested that the immune milieu of chronic rejection was consistent across patient samples. **(A)** Principal component analysis (PCA) of median signal intensity for each IMC marker revealed that Principal Component (PC) 1 explained 51.47% of the variance across markers. **(B)** The relative contributions of each marker for each component demonstrate that each of the immune markers contribute to PC1, rather than one specific immune population. **(C)** PC1 accounted for most differences between liver with no rejection and chronic rejection (Wilcoxon Rank Sum test, p=0.000036). **(D)** Based on a logistic regression on PC1, regions of interest from chronic rejection were correctly modeled with at least 75% probability, with only one outlier from liver with no rejection and chronic rejection samples failing to group within the model. PCA was also performed using immune meta-cluster proportions. **(E)** PC1 explained 36.16% of the variance across immune meta-clusters. **(F)** The relative contributions of each immune meta-cluster in each PC are shown, highlighting that multiple immune subpopulations contribute to PC1. **(G)** PC1 accounted for most differences between liver with no rejection and chronic rejection (Wilcoxon Rank Sum test, p=0.0000023). **(H)** Based on a logistic regression on PC1, regions of interest were modeled with the same accuracy as in **(D)** but with two chronic rejection samples falling close to the 50% cut point.

Another of the major advantages of using highly dimensional single cell datasets involves the ability to use these data to create predictive algorithms of clinical outcomes. Indeed, blood-based CyTOF and single cell IMC data have been applied to complex modeling algorithms to predict clinical outcomes in other disease processes, including gestational age in pregnancy (15, 32, 33). For this phase of our study, we again compared the broad groups of NR and CR, to determine how similar or different immune profiles are within each clinical condition. First, median marker signal per ROI were examined. Coordinates from tSNE plots of each ROI were examined using box plots, which suggested that similarities existed across patients with the CR and NR cohorts within the immune markers that we considered for analysis (**Figure S3**). To explore these dimensions further, PCA was conducted to similarly reduce dimensionality and capture distinct separation observed in the tSNE plots while simultaneously preserve interpretability of features and identify potential relationships between IMC data and association with CR. PCA revealed that principal component (PC) 1 explained 51.47% of the variance across immune markers, and this component was different between CR and NR (p=0.000036, **Figures 6A, C**). Next, the specific contribution of each immune marker was examined for each PC, and PC1 had similar contributions from CD20, CD3, CD45, CD66, CD8, and HLA-DR (**Figure 6B**). A logistic regression model based on PC1 demonstrated that CR was correctly modeled with at least 75% probability, with only one outlier in each of the NR and CR ROI (**Figure 6D**).

Based on the success of PCA and logistic regression modeling using median signal intensity rather than single cell data, further modeling was explored using immune cell meta-clusters for CR and NR. Similar results were obtained, with PC1 explaining 36.16% of the variance across immune meta-clusters (**Figure 6E**), with statistical difference between CR and NR (p=0.000023, **Figure 6G**). Most of the immune meta-clusters are represented in PC1, with Macrophage-2 and CTL-2 being most dominant (**Figure 6F**). Logistic regression modeling of PC1 determined that CR was correctly modeled with the immune markers, with only one outlier in the NR ROI and two in the CR (**Figure 6H**).

## Discussion

Since the introduction of clinical solid organ transplantation in the 1950s, tissue-based diagnosis of rejection has relied on histopathological evaluation and interpretation by experienced, specialized pathologists. Difficulties in assessing alloimmunity at the tissue level have limited the identification of pathogenic immune subsets and further development of personalized biomarkers and treatments of rejection. In this study, IMC was explored as a novel single cell technique to characterize alloimmunity in existing, FFPE clinical LT specimens. By using a pilot panel of 10 IMC markers and analysis with computational biology approaches, we highlight the potential for this technique to deeply characterize the immune microenvironment within a rejecting allograft at a single cell level. First, this study expands prior work illustrating that IMC produces reliable, high quality staining on FFPE tissue sections, regardless of vintage and storage, in a similar time frame as traditional immunohistochemistry but without the challenges of autofluorescence and background staining (15–17). Second, we established that our single cell segmentation pipeline enables highly dimensional, qualitative and quantitative analysis of immune subpopulations involved in areas of inflammation. Third, we demonstrated that spatial relationships and interactions between immune subsets can be quantified. Finally, we determined that single cell datasets generated using IMC can be incorporated into models designed to stratify IMC features by clinical outcomes with high reliability. Collectively, these findings suggest that IMC, which has the potential to be expanded as more immune markers are validated to include >30 antibodies for use on a single tissue section, has the potential to be a transformative and paradigm shifting novel approach to the diagnosis and management of rejection in clinical solid organ transplantation. IMC offers a new capability to quantify severity and complexity of inflammation, monitor graft-infiltrating alloreactive lymphocytes during immunosuppression withdrawal trials, identify new drug targets, and develop predictive models that can be reliably associated with clinical outcomes.

Using a pilot panel based on 10 existing and validated markers, IMC enabled identification of 11 unique immune subpopulations across the entire study cohort. Among these, only one was unique to CR, Macrophage-1. Also, not all subpopulations expressed CD45, which is consistent with prior work in clinical LT biopsies using multiplexed immunohistochemistry and IMC (15, 19–21, 34). While neutrophils and Other Leukocytes (CD45+ cells) were rare overall at <10% of the immune cell microenvironment, NR had a higher proportion of these populations when compared to CR (**Figure 4**). These findings correlate with prior work examining neutrophil to lymphocyte ratio in the blood of LT recipients during episodes of rejection, where patients with TCMR had lower scores, and thus lower proportions of neutrophils, when compared to patients without rejection (35). While no studies, to our knowledge, have examined the role of neutrophils in rejection episodes at the tissue level, prior work has established a role for neutrophils during hepatic ischemia-reperfusion injury and fibrogenesis in humans (36, 37). In our study we excluded liver grafts with extensive fibrosis to reduce the potential for fibrogenesis to alter the immune microenvironment. A study examining a large population of liver biopsies with varying degrees of fibrosis demonstrated that as fibrosis progressed to F3/F4, a higher proportion of neutrophils was present (37). Our observation that neutrophils were rare during CR supports our study design, where patients with advanced fibrosis were excluded. Four immune subsets were more prominent in CR versus NR: Macrophage-2 (p<0.01), CTL-2 (p<0.01), Other T-cell-1 (CD45+CD3+; p<0.05), and Other T-cell-2 (CD45+CD3+; p<0.05) (**Figure 4**). Increases in T-cell populations during CR were expected, as they represent a dominant mediator of alloimmunity (38). We were unable to further characterize the key features of these T-cell subsets, including the role for CD4+ effector or CD4+Foxp3+ regulatory T-cells, which is a limitation of our pilot IMC antibody panel. However, IMC still enabled a highly granular and quantitative exploration of T-cell subpopulations using relatively few conventional immune markers (CD45RA, CD45, CD3, and CD8), resulting in identification of five distinct T-cell subsets. As more IMC-compatible antibodies become available, addition of markers for potentially important T-cell subsets such as CD3+CD4+Foxp3+ regulatory T-cells will enable further phenotyping of these cells within the tissue, providing the opportunity to explore mechanisms of alloimmunity in more detail.

Two distinct macrophage populations were observed in CR, which may represent Kupffer cells and inflammatory macrophages. The relative abundance of these two macrophage populations has been implicated in a variety of pathological states within the human liver, including rejection (39–41). CD68 expression is common to both Kupffer cells, liver-specific macrophages which share an embryonic origin with hepatocytes, and inflammatory macrophages, which are bone marrow derived (42). Interestingly, Macrophage-2 was more prominent in CR, while Macrophage-1 was not present in NR. A prior study examining CD68+ in nine sex mismatched donor and recipient pairs with CR post-LT determined that CD68+ cells were prominent and largely of recipient origin, suggesting that Macrophage-1 are recipient derived inflammatory macrophages (43). If this is true, then expansion of Macrophage-2, which could represent donor derived Kupffer cells, would suggest that a CR is at least in part driven by donor-derived immune populations. Moving forward, further phenotyping of these CD68+ populations using expanded IMC panels and complementary techniques such as single cell RNASeq will expand our understanding of their potential pathogenic role in CR (44). Importantly, several drugs known to target intrahepatic macrophages, including cenicriviroc, are being investigated in ongoing clinical trials (39). Our data suggest that these therapies could be explored for efficacy in downregulating macrophage-mediated alloimmunity during rejection episodes in LT.

Deeper examination of significant spatial relationships between immune subsets using neighborhood analysis revealed the complexity of the immune microenvironment of CR, even with a somewhat limited sample size of 18 patients. Immune subpopulations did not interact with each other in neighborhood analysis in NR (**Figure 5B**), and this was confirmed by visualization on NR ROI, where rare, spatially separated immune cells were frequent (**Figures 1**, **2**, **4**, **5**). This suggests a state of immune equilibrium in NR, which is supported by recent single cell RNA sequencing studies illustrating that intrahepatic immune subsets in normal human liver prominently express homeostatic genes rather than those involved in activation (44–46). The immune microenvironment of NR as characterized by IMC has implications as an important reference point when attempting to establish operational tolerance post-LT, as surveillance biopsies are a cornerstone of these studies to rule out subclinical rejection during immunosuppression withdrawal (34, 38, 47). Within CR, several immune subpopulations had strong, significant interactions within a subset, including Macrophage-2, CTL-2, and Other T-cell 1 (**Figure 5**). This suggests the presence of immune proliferation and an active, smoldering inflammatory response despite the chronicity of disease. Strong interactions were also observed between populations, including CTL-2 and Macrophage-2, CTL-1 and CTL-2, and B-cell with Other T-cell-1, which were confirmed by visualizing specific subsets on tissue sections (**Figure 5C**). These findings are supported by prior work examining tissue-specific immune interactions in clinical liver tissue, where semi-quantitative approaches and multiplexed immunofluorescence identified interactions between macrophages and CD3+ T-cells as well as an ‘immune synapse’ between HLA-DPB1+ cells and protein tyrosine phosphatase receptor type C-positive leukocytes during TCMR post-LT in the pediatric iWITH study (34, 48). Moving forward, quantitative neighborhood analysis of IMC data during rejection episodes has potential clinical value in evaluating response to therapeutic intervention, especially when evaluating immunotherapies designed to target specific subpopulations.

While single cell applications are still emerging in the field of solid organ transplantation, prior studies in other disease states have correlated highly dimensional mass cytometry datasets with clinical outcomes (12). Gaudilliere et al. examined blood samples using CyTOF and associated immune phenotypes with post-operative recovery as well as gestational age in pregnancy (32, 33). By examining an 18 patient training cohort and 10 patient validation cohort using Elastic Net regression modeling of immune subsets in peripheral blood, a model was constructed that predicted gestational age with high accuracy (R=0.62, p=2.4x10^-4^) (32). More recently, the Bodenmiller group published a detailed analysis of breast cancer using IMC, with novel histologic subtypes and cellular neighborhoods correlating with survival outcomes (15). Similar to these prior studies, examination of a modest and clinically heterogenous population of LT recipients with CR using IMC and PCA with logistic regression resulted in a highly consistent immune phenotype associated with CR, using both raw signal intensity per marker in each ROI and single cell meta-cluster data (**Figure 6**). Importantly, PC-1 represented most of the variability and was composed of multiple immune markers, rather than a single dominant population as shown in the remaining PCs. This is supported by quantification and spatial analysis of immune populations in CR (**Figures 4**, **5**), where macrophages, T-cells, and other lymphocytes including B-cells were upregulated and/or had significant interactions on neighborhood analysis. When examined using logistic regression modeling, PC-1 was associated with CR with >75% probability, with only 1-2 ROI outlier depending on the data being analyzed (**Figure 6**). It is possible that with a larger patient cohort, validation dataset, and expanded IMC marker panel, machine learning algorithms can be developed to accurately predict clinical trajectories during rejection, including steroid resistance, impending CR, and candidacy for immunosuppression withdrawal.

There are limitations to this study. Patients were studied from a single center, retrospectively, with different immunosuppressive regimens, frequency of prior rejection episodes, and intervals between first and second LT, and diagnosis of CR is based on pathologic assessment using consensus criteria but without a discrete scoring system (22). We are still optimizing our protocol for use in needle core biopsies, so we were not able to use stable post-LT biopsies as a reference group in this pilot study. However, our NR normal liver was confirmed to be histologically similar to stable post-LT liver biopsies by an experienced liver transplant pathologist. We did not have access to fresh tissue or blood samples from these patients to correlate immune phenotypes using complementary techniques such as single cell RNASeq, CyTOF, or plasma proteomics. Similarly, we were limited by the selection of validated immune marker antibodies for use in human FFPE tissue using IMC and plan to expand on these studies as additional markers become available. Specifically, relatively few T-cell markers were available for analysis, such as CD4, Foxp3, CD28, Granzyme B, etc, limiting identification of the role of potentially important subsets including regulatory T-cells. Also, as stated in the results, while IMC signal intensity for a specific marker can be quantified within an ROI and across samples prepared using the same tissue type, during the same experiment, signal intensity between different antibody markers in IMC cannot be interpreted to indicate relative antigen density as one might consider with traditional flow cytometry or CyTOF. From a technical perspective, single cell segmentation algorithms have the potential to capture markers from an adjacent cell, thereby skewing results; however, over 30,000 immune cells were analyzed, and all subsets identified had marker expression profiles that matched established immune populations. Nonetheless, our segmentation and Phenograph analysis pipeline may have resulted in identification of immune meta-clusters, particularly those with similar markers, that may not exhibit substantial functional differences.

In conclusion, this study establishes a novel, IMC-based approach to quantify alloimmunity in clinical biopsy specimens in transplant recipients. Creation of a single cell dataset with spatial information enabled identification of 11 distinct immune populations involved in CR and highlighted the complexity of the immune microenvironment within the allograft. IMC staining can be completed on existing clinical FFPE tissues slides in a matter of hours, and our study suggests that this approach may be rapidly adapted to deeply characterize rejection episodes, identify new therapeutic targets, and develop predictive models of immune-mediated disease progression in solid organ transplantation.

## Data Availability Statement

The original contributions presented in the study are included in the article/**Supplementary Material**, further inquiries can be directed to the corresponding author.

## Ethics Statement

The studies involving human participants were reviewed and approved by Institutional Review Board, University of Southern California. Written informed consent from the participants’ legal guardian/next of kin was not required to participate in this study in accordance with the national legislation and the institutional requirements.

## Author Contributions

Involved in the conception or design of the work, JE, OA, and SA. Data acquisition and statistical analysis, NU, CG, CM, JH, RS, AB, and SC. Analysis and interpretation of data, JE, NU, JL, LS, SA, RK, OA, and YG. Drafted the article, NU and JE. Critically revised the article, all contributing authors. All authors contributed to the article and approved the submitted version.

## Funding

JE was supported by an Investigator Research Supplement from the National Heart, Lung, and Blood Institute of the National Institutes of Health under award number R01 HL141857-01 and a K08 Award from the National Cancer Institute under award number K08 CA245220-01. We also received funding for this project from a USC Dean’s Pilot Grant, USC Research Center for Liver Diseases, and Children’s Hospital-Los Angeles.

## Conflict of Interest

The authors declare that the research was conducted in the absence of any commercial or financial relationships that could be construed as a potential conflict of interest.

## Publisher’s Note

All claims expressed in this article are solely those of the authors and do not necessarily represent those of their affiliated organizations, or those of the publisher, the editors and the reviewers. Any product that may be evaluated in this article, or claim that may be made by its manufacturer, is not guaranteed or endorsed by the publisher.
