# Supplementary material for: Adaptation of Imaging Mass Cytometry to Explore the Single Cell Alloimmune Landscape of Liver Transplant Rejection
Source: Front Immunol. 2022 Mar 31;13:831103. doi: 10.3389/fimmu.2022.831103 (PMC9009043; doi:10.3389/fimmu.2022.831103)
Supplement: Supplementary file 1 [file Table_1.docx]

Supplementary Material

| Patient | Age at 2nd Transplant(years) | Sex | Race/ Ethnicity | Original Diagnosis | First Graft Type | Rejection episodes after First LT (N) | Rejection classification on Biopsy | Time from transplant to first rejection (months) | Treatments of rejection episodes | Interval between 1^st^ and 2^nd^ transplant (months) |
| --- | --- | --- | --- | --- | --- | --- | --- | --- | --- | --- |
| 1 | 1 | M | White | ALF | Split Deceased | 1 | ACR | 0.4 | IVIG  ATG  steroids | 0.7 |
| 2 | 9 | F | Hispanic | ALF | Split Deceased | 3 | ACR x 2  CR x 1 | 10.1 | Steroids | 18 |
| 3 | 11 | M | Hispanic | Biliary atresia | Split Deceased | 3 | ACR x 1  CR x 2 | 10 | Steroids | 127.5 |
| 4 | 15 | F | Asian | Metabolic | Whole Deceased | 2 | ACR x 2  CR x 1 | 5 | Steroids | 99.3 |
| 5 | 23 | F | Hispanic | Biliary atresia | Split Deceased | 5 | CR x 5 | 261.7 | Steroids | 288 |
| 6 | 23 | M | White | ALF | LDLT  (R. Lobe) | 2 | CR x 2 | 73.7 | Steroids | 86.4 |
| 7 | 32 | F | Hispanic | ALF | Whole Deceased | 7 | ACR x 3  CR x 4 | 65.9 | Steroids | 144 |
| 8 | 32 | M | White | GSD | Whole Deceased | 1 | CR x 1 | 197.8 | Steroids | 199.2 |
| 9 | 34 | F | Hispanic | Wilson Disease | Whole Deceased | 4 | CR x 4 | 120.9 | Steroids | 174 |
| 10 | 35 | F | Hispanic | AIH | Whole Deceased | 8 | ACR x 2  CR x 6 | 5.5 | Steroids | 189.5 |
| 11 | 47 | F | White | HBV | Whole Deceased | 3 | ACR x 1  CR x 2 | 1.1 | OKT3  Steroids | 7.4 |
| 12 | 50 | M | Hispanic | HBV | Whole Deceased | 3 | ACR x 2  CR x 1 | 6.6 | Steroids | 51.3 |
| 13 | 50 | M | White | HCV/ALD | Whole Deceased | 3 | ACR x 1  CR x 2 | 0.9 | Steroids | 7.2 |
| 14 | 51 | M | Hispanic | HCV/HCC | Whole Deceased | 2 | CR x 2 | 8.5 | Steroids | 13.1 |
| 15 | 53 | M | White | ALD | Whole Deceased | 1 | CR x 1 | 30.2 | Steroids | 32.4 |
| 16 | 54 | M | Hispanic | HCV/ALD | Whole Deceased | 2 | ACR x 1  CR x 1 | 0.3 | Steroids | 9.2 |
| 17 | 55 | M | Hispanic | ALD | Whole Deceased | 2 | ACR x 1  CR x 1 | 6.1 | Steroids | 8.7 |
| 18 | 56 | M | Caucasian | HCV | Whole Deceased | 2 | ACR x 1  CR x 1 | 0.9 | Steroids | 1.3 |

**Supplementary Table 1: Summary of patient characteristics in CR group.**

ALF: acute liver failure, GSD: glycogen storage disorder, AIH: autoimmune hepatitis. HBV: Hepatitis B Virus, HCV: Hepatitis C Virus. ALD: Alcohol-related Liver Disease, HCC: hepatocellular carcinoma, ACR: acute cellular rejection, CR: chronic rejection, ATG: anti-thymocyte globulin

| Target | IMC Clone/ dilution | IMC Supplier | IHC Clone/ dilution | IHC Supplier |
| --- | --- | --- | --- | --- |
| CD45 | CD45-2B11 (1:100) | Fluidigm | X16/99 (RTU) | Leica Biosystems Inc |
| CD68 | KP1  (1:200) | Fluidigm | 514H12 (RTU) | Invitrogen |
| CD20 | H1  (1:200) | Fluidigm | L26  (RTU) | Cell Marque |
| CD8a | C8/144B  (1:200) | Fluidigm | 4B11 (RTU) | Leica Biosystems Inc |
| CD45RA | HI-100  (1:50) | Fluidigm | HI-100 (1:300) | Biolegend |
| Collagen Type I | goat polyclonal  (1:250) | Fluidigm | goat polyclonal (1:50) |  |
| CD3 | poly, C-Terminal  (1:150) | Fluidigm | LN10 (RTU) | Leica Biosystems Inc |
| CD66a | CD66a-B11  (1:50) | Fluidigm | ASL-32 (1:100) | Biolegend |
| HLA-DR | YE2/36 HLK  (1:50) | Fluidigm | LN3 (1:2000) | Invitrogen |

**Supplementary Table 2: Antibody clones and suppliers.**

| Subpopulation | Cluster ID | Number of Cells |
| --- | --- | --- |
| B-Cell | 24 | 1420 |
| Cytotoxic T-Cell 1 | 16 | 2708 |
| Cytotoxic T-Cell 2 | 8 | 5553 |
| HLADR | 26 | 1045 |
| Macrophage 1 | 22 | 1433 |
| Macrophage 2 | 3 | 7135 |
| Naïve T-Cell | 23 | 1381 |
| Neutrophil | 25 | 1238 |
| Other Lymphocyte | 28 | 768 |
| Other T-Cell 1 | 17 | 2570 |
| Other T-Cell 2 | 9 | 5395 |
| Non-Immune Cell | 1, 2, 4-7, 10-15, 18-21, 27, 29 | 78599 |

**Supplementary Table 3: Phenograph Subpopulation Key.**


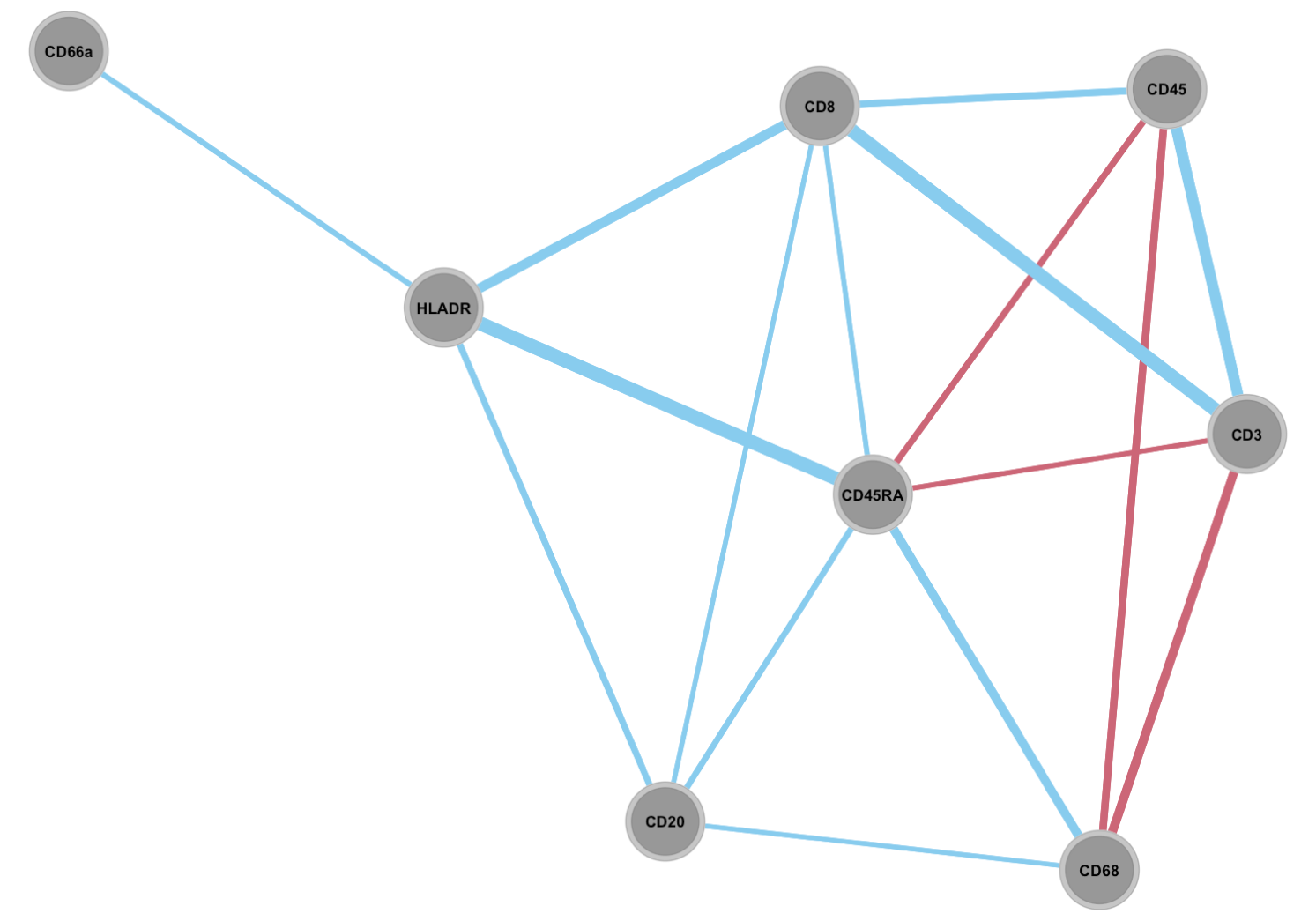


**Supplementary Figure 1: Correlation Network of IMC Markers.** Network showing strength and direction of correlations in immune markers. Blue indicates positive associations while red indicates negative associations. Thickness of connecting edges is proportional to strength of correlations. Weak correlations less than ±0.10 not shown.

**
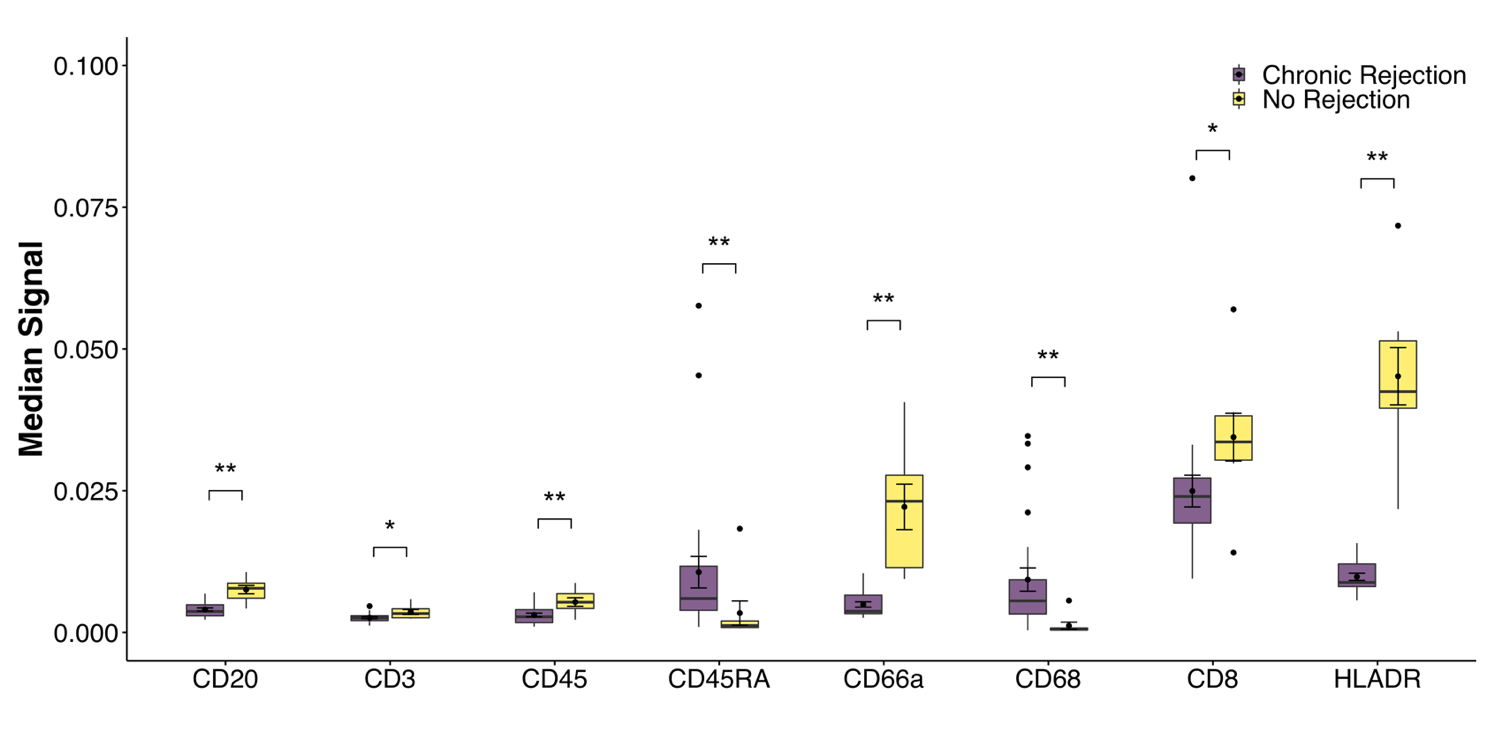
**

**Supplementary Figure 2: Highly dimensional, single-cell immune phenotyping of human liver tissue with IMC.** Median signal intensity per region of interest for immune markers in IMC panel in each patient cohort (Chronic Rejection (N=18): purple, No Rejection (N=5): yellow) is shown. *p<0.05, ** p<0.01 for pairwise comparisons.

**
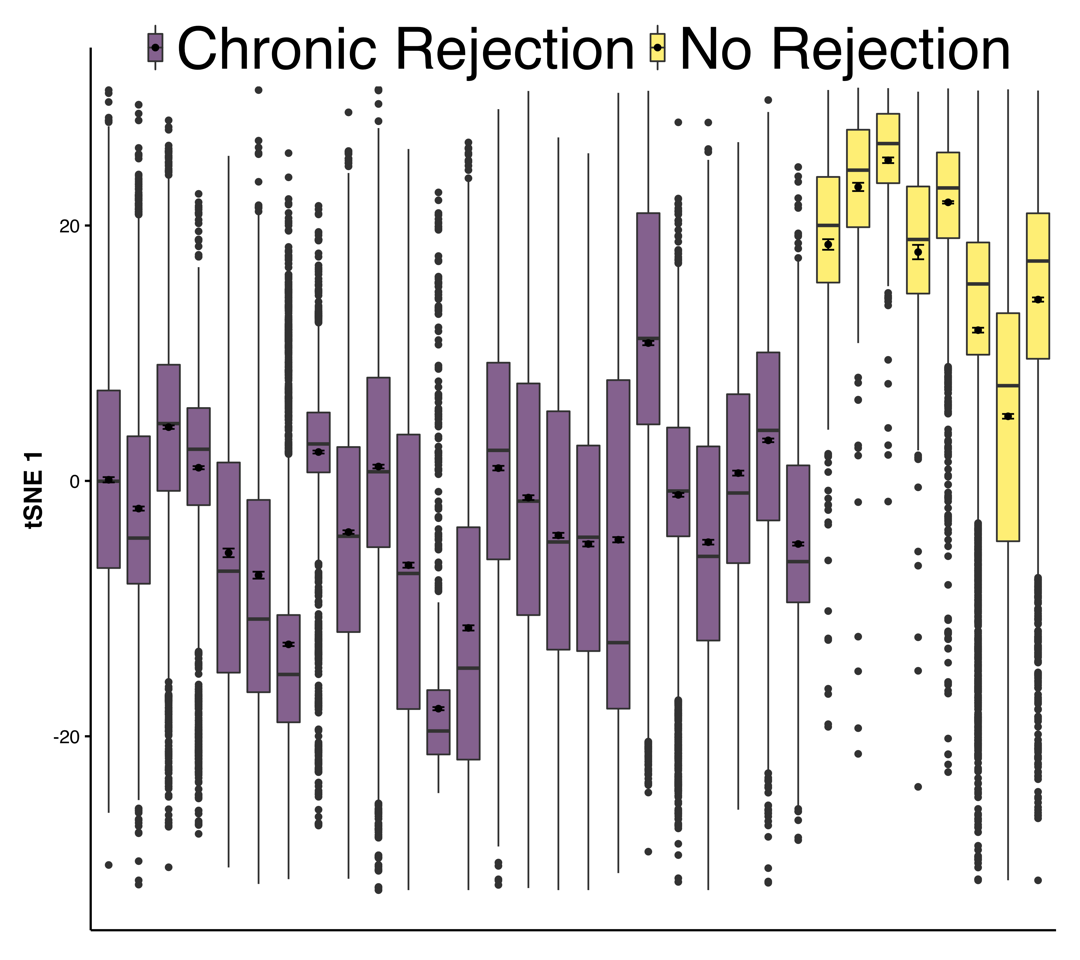
**

**Supplementary Figure 3: Coordinates of First tSNE Component Distinguishes Chronic Rejection and No Rejection.** Median signal intensity per region of interest for immune markers in IMC panel in each patient cohort (Chronic Rejection (N=18): purple, No Rejection (N=5): yellow) is shown. *p<0.05, ** p<0.01 for pairwise comparisons.
